# Comparative Genomics of Pathogens Causing Brown Spot Disease of Tobacco: *Alternaria longipes* and *Alternaria alternata*

**DOI:** 10.1371/journal.pone.0155258

**Published:** 2016-05-09

**Authors:** Yujie Hou, Xiao Ma, Wenting Wan, Ni Long, Jing Zhang, Yuntao Tan, Shengchang Duan, Yan Zeng, Yang Dong

**Affiliations:** 1 Faculty of Life Science and Technology, Kunming University of Science and Technology, Kunming, Yunnan, China; 2 Longrun Pu-erh Tea Academy, Yunnan Agricultural University, Kunming, Yunnan, China; 3 College of Life Science and Technology, Huazhong University of Science and Technology, Wuhan, Hubei, China; 4 State Key Laboratory of Genetic Resources and Evolution, Kunming Institute of Zoology, Chinese Academy of Science, Kunming, Yunnan, China; 5 Biological Big Data College, Yunnan Agricultural University, Kunming, Yunnan, China; The University of Wisconsin - Madison, UNITED STATES

## Abstract

The genus *Alternaria* is a group of infectious/contagious pathogenic fungi that not only invade a wide range of crops but also induce severe allergic reactions in a part of the human population. In this study, two strains *Alternaria longipes* cx1 and *Alternaria alternata* cx2 were isolated from different brown spot lesions on infected tobacco leaves. Their complete genomes were sequenced, *de novo* assembled, and comparatively analyzed. Phylogenetic analysis revealed that *A*. *longipes* cx1 and *A*. *alternata* cx2 diverged 3.3 million years ago, indicating a recent event of speciation. Seventeen non-ribosomal peptide synthetase (NRPS) genes and 13 polyketide synthase (PKS) genes in *A*. *longipes* cx1 and 13 NRPS genes and 12 PKS genes in *A*. *alternata* cx2 were identified in these two strains. Some of these genes were predicted to participate in the synthesis of non-host specific toxins (non-HSTs), such as tenuazonic acid (TeA), alternariol (AOH) and alternariol monomethyl ether (AME). By comparative genome analysis, we uncovered that *A*. *longipes* cx1 had more genes putatively involved in pathogen-plant interaction, more carbohydrate-degrading enzymes and more secreted proteins than *A*. *alternata* cx2. In summary, our results demonstrate the genomic distinction between *A*. *longipes* cx1 and *A*. *altenata* cx2. They will not only improve the understanding of the phylogenetic relationship among genus *Alternaria*, but more importantly provide valuable genomic resources for the investigation of plant-pathogen interaction.

## Introduction

*Alternaria* is a genus of ubiquitous fungi that includes saprobic, endophytic and pathogenic species associated with a wide variety of hosts [1]. The members of *Alternaria* infect a remarkable range of plants, including citrus, pistachio, apple, pear, tobacco, tomato, and beans, causing devastating plant diseases and resulting in considerable loss of agricultural yield.

**Fig 1:**
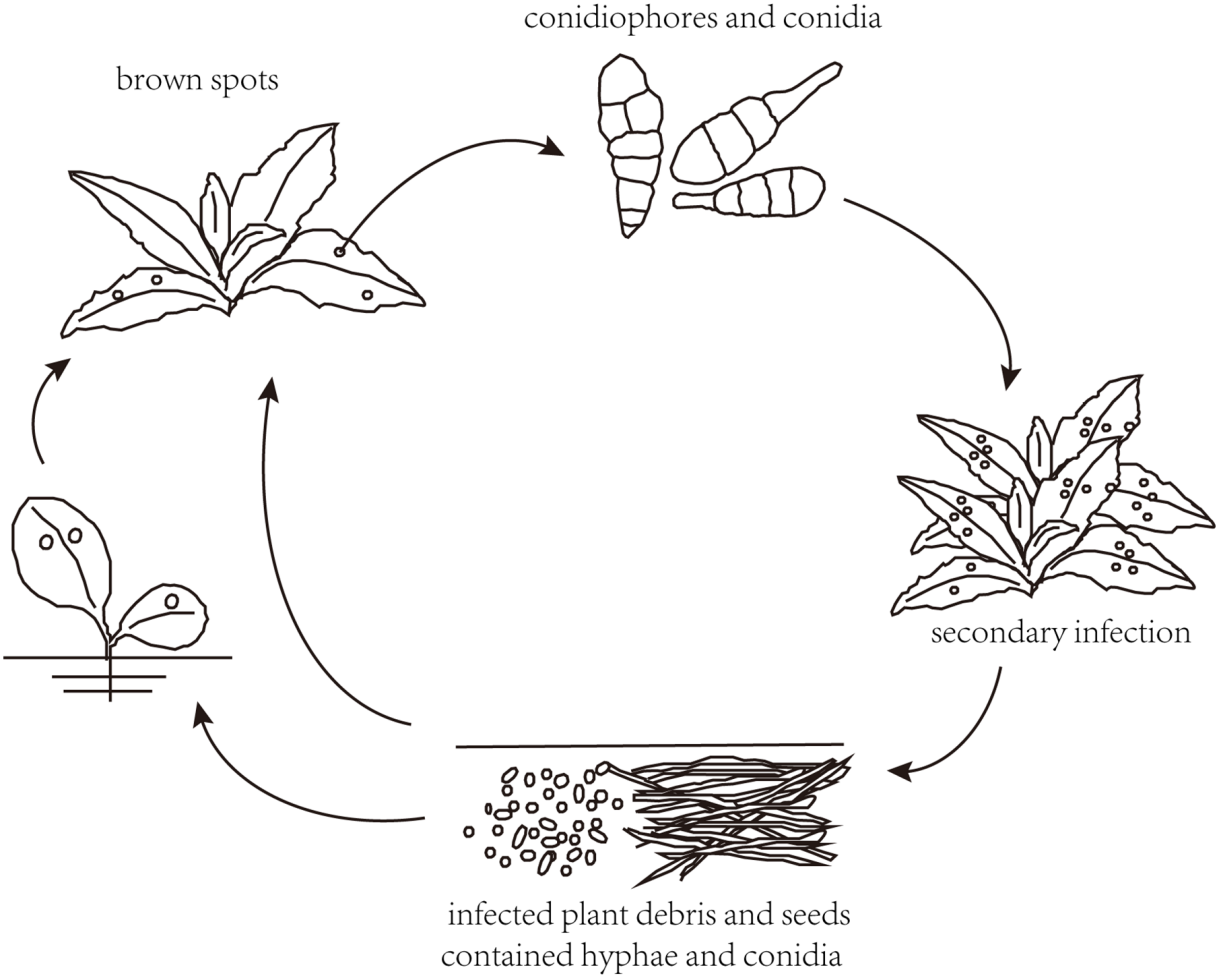
Saprophytic life cycle of *Alternaria* invading its host plants.

Brown spot disease is one of the most destructive leaf spot diseases caused by *Alternaria* on various crops. It has been reported that the members of the genus *Alternaria* are major fungal pathogens that infect tobacco leaves, and give rise to the formation of brown spot [2]. After the plant enters the growing stage and the climate becomes suitable, the conidia of *Alternaria* will spread very rapidly in the field (Fig 1). They germinate and infect crop leaves or fruits, which not only causes great loss of agricultural production, but the produced mycotoxins present in agricultural products also threatens the health of humans and animals.

However, there are always confusions about species identification in the genus *Alternaria*. Some studies suggested that some host-specific toxins (HSTs) producing pathogens in *Alternaria* (*A*. *mali*, *A*. *citri*, *A*. *kikuchiana*, *A*. *longipes* and *A*. *alternata* f. sp. *lycopersici*) look similar in conidial morphology and should be interpreted as intraspecific variability of *A*. *alternata* [3–5]. However, other researchers argued that *A*. *longipes* and *A*. *alternata* are different species that can be distinguished by morphological species concepts [6], molecular [7] and chemical methods [8]. They argued that the continuing use of the name *A*. *alternata* for *A*. *longipes* is unwarranted and that pathotypes should not be used [8].

Recent studies have suggested that *Alternaria* spp. produce non-host specific toxins (non-HSTs) (e.g., tenuazonic acid (TeA), alternariol (AOH), alternariol monomethyl ether (AME), brefeldin A, tentoxin, zinniol) [9] and host-specific toxins that produced by a gene cluster usually residing on one or several conditionally dispensable chromosomes (CDCs) [1, 10]. Two major toxins, AT-toxin and TeA, are involved in the onset of tobacco brown spot disease [11]. TeA is a nonspecific toxin, whereas AT-toxin is considered as a HST to *Nicotiana tabacum*. In contrast to what is known about the chemical structures and properties of other *Alternaria* HSTs [12], such as AK-toxin [13], ACT-toxin [14] and AM-toxin [15], no detailed information is available for AT toxin, while researchers proposed that they induced programmed cell death in tobacco [16]. Some reports also found that TeA and other low molecular compounds could cause a faded green halo around the invasion site, and finally gave rise to the brown spot.

Although the symptoms of brown spot are usually associated with the action of *A*. *alternata* or *A*. *longipes*, their pathogenicity mechanisms including toxin biosynthesis pathways, secondary metabolism and secretomes are still unclear and need further investigation. In addition, most research on *Alternaria* would greatly benefit from the information of a reference genome.

Large-scale genome sequencing and comparative genome analysis can help to identify shared and unique pathogenicity genes in closely-related fungal species. In this study, two isolates named CX1 and CX2 were isolated from different infected tobacco cultures by separation of single fungal spores. CX1 and CX2 were separated from typical brown spot lesions on tobacco leaves and brown spots lesions on sunburned tobacco leaves, respectively. Through the whole genome sequencing and phylogenetic relationship analysis based on co-linear sequences, CX1 and CX2 were identified as *A*. *longipes* and *A*. *alternata*, respectively. Accordingly, we renamed CX1 to *A*. *longipes* cx1 and CX2 to *A*. *alternata* cx2. Moreover, a variety of comparative genomic analyses revealed differences between the *A*. *longipes* cx1 and *A*. *alternata* cx2 genomes including genes putatively involved in non-HSTs biosynthesis, pathogen-plant interaction, cell wall integrity and secreted proteins.

## Results and Discussion

### Strain identification based on ITS

**Fig 2:**
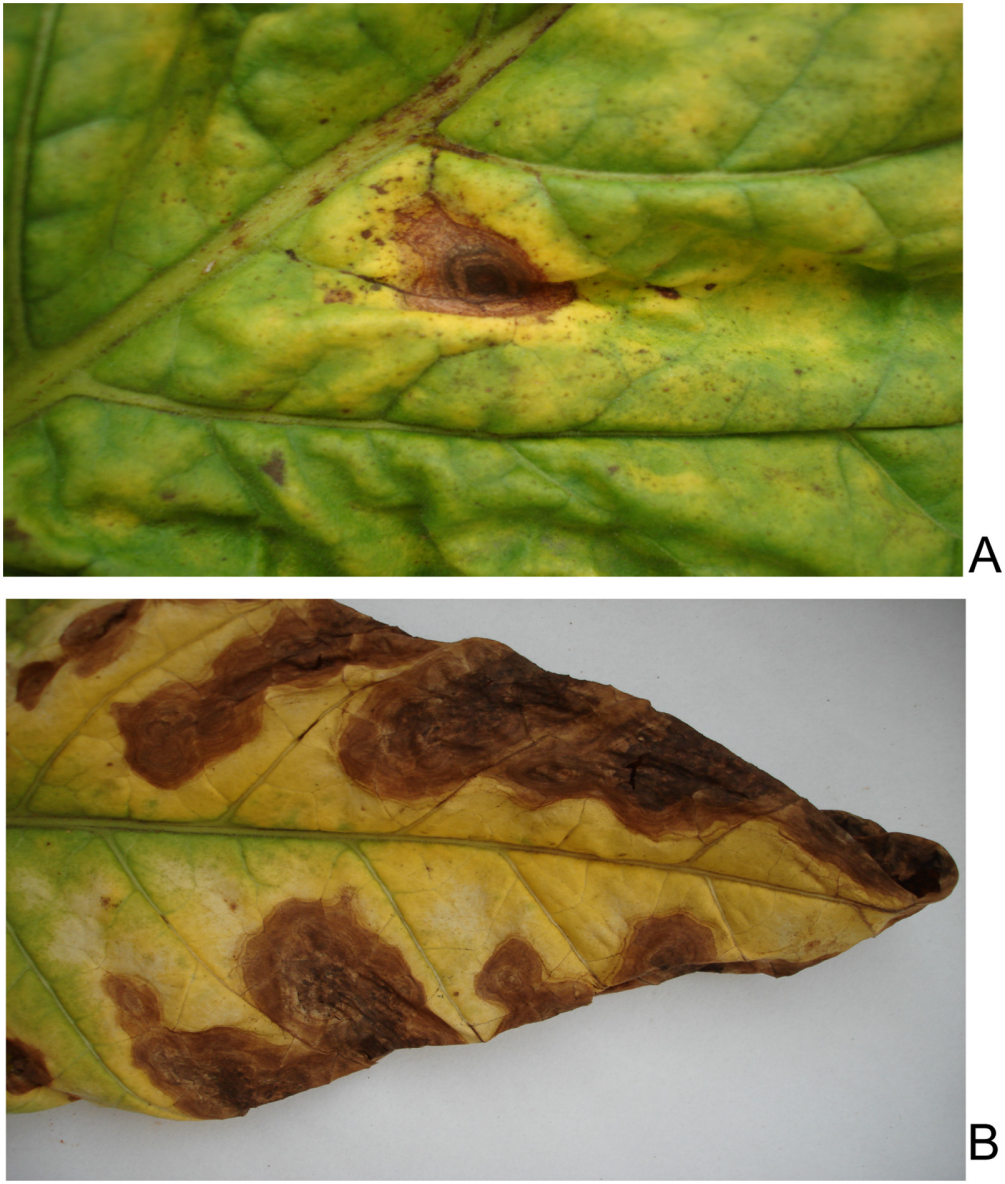
Tobacco leaves showing different brown spot symptoms. (A) Typical brown spot lesions on tobacco leaves, from which CX1 was isolated. (B) Brown spots lesions on sunburned tobacco leaves, which served to isolate CX2.

**Fig 3:**
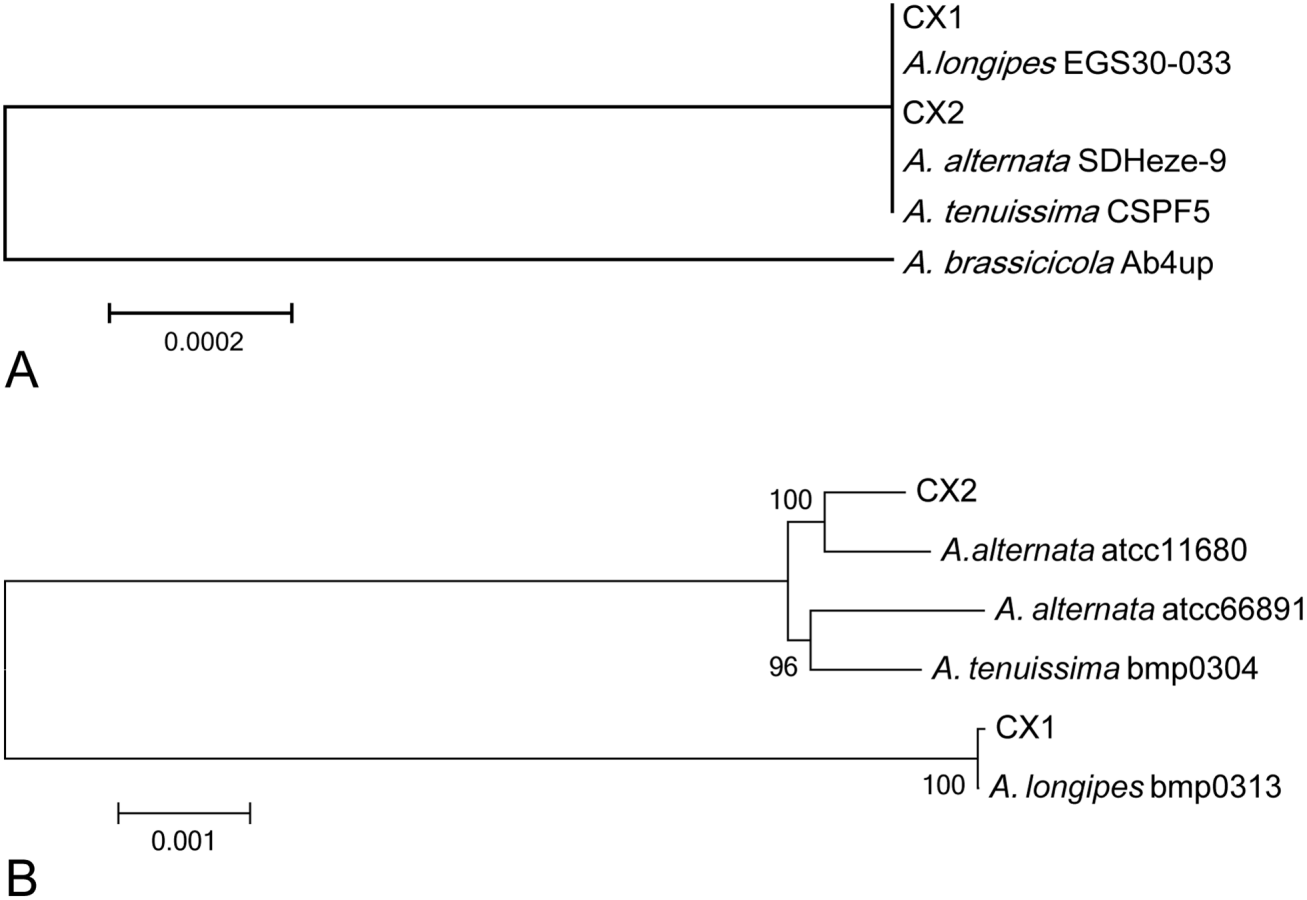
Phylogenetic relationship between CX1 and CX2. (A) Phylogenetic relationship constructed by MEGA5 based on ITS sequences of CX1, CX2, *A*. *longipes* EGS30-033, *A*. *alternata* SDHeze-9, *A*. *tenuissima* CSPF5 and *A*. *brassicicola* Ab4UP. (B) Phylogenetic relationship constructed by MEGA5 based on large co-linear sequence among CX1, CX2, *A*. *alternata* atcc11680, *A*. *longipes* bmp0313 and *A*. *tenuissima* bmp0304.

Two strains named CX1 and CX2 were isolated from typical brown spots lesions on tobacco leaves and sunburned tobacco leaves (Fig 2) by separating single fungal spores, respectively. A phylogenetic tree based on ITS sequences was built among CX1, CX2, *A*. *longipes* EGS30-033 (accession: AY751457.1), *A*. *alternata* SDHeze-9 (accession: KT238888.1), *Alternaria tenuissima* CSPF5 (accession: KU508797.1) and *Alternaria brassicicola* Ab4UP (accession: KF542552.1) from NCBI (Fig 3A), showing that CX1, *A*.*longipes* EGS30-033, CX2, *A*. *alternata* SDHeze-9 and *A*. *tenuissima* CSPF5 are clustered in the same clade.

Previous researchers demonstrated that the ITS variability within the genus is relatively limited. A number of taxa inhabiting particular plant species, such as *A*. *longipes*, *A*. *mali* and *A*. *tenuissima* cannot reliably be distinguished from *A*. *alternata* using this method [17]. Elisabeth [18] argued that isolates of the *A*. *alternata*, *A*. *tenuissima*, and *Alternaria arborescens* species-groups could not be further resolved by ITS. In this study, the ITS sequences in CX1 and CX2, differed by only two bases (S1 Text), confirming that species separation between CX1 and CX2 cannot be done by comparison of ITS sequences.

Therefore, a phylogenetic tree based on large co-linear sequences (refer to methods and S2 Text) was constructed by MEGA5 among CX1, CX2, *A*. *alternata* atcc11680, *A*. *alternata* atcc66891, *A*. *longipes* bmp0313 and *A*. *tenuissima* bmp0304 from *Alternaria* Genomes Database [19] (Fig 3B). This analysis demonstrated that CX1 is more closely related to *A*. *longipes* while CX2 and *A*. *alternata* have a closer genetic relationship. Based on our result, CX1 and CX2 were determined as *A*. *longipes* and *A*. *alternata*, respectively, and renamed to *A*. *longipes* cx1 and *A*. *alternata* cx2.

### General features of the *A*. *longipes* cx1 and *A*. *alternata* cx2 genomes

The genomes of *A*. *longipes* cx1 and *A*. *alternata* cx2 were sequenced on the Illumina HiSeq 2500 platform using a whole genome shotgun approach. This generated a total of 27.75 Gb raw sequences for *A*. *longipes* cx1, and 17.99 Gb raw sequences for *A*. *alternata* cx2 as 100 bp paired-end short reads. Quality-control filters removed adapter sequences, regions of low base-call quality, and regions of low sequence complexity. All *17-mer* sequences were then extracted from each library. The *17-mer* analysis showed that both genomes of *A*. *longipes* cx1 and *A*. *alternata* cx2 had low heterozygosity (S1 Fig). The genome sizes of *A*. *longipes* cx1 and *A*. *alternata* cx2 were estimated to be 39.99 Mb and 37.40 Mb, respectively. The sequencing depth was calculated to be about 694 × and 481 × for *A*. *longipes* cx1 and *A*. *alternata* cx2, respectively.

The total assembly sizes of *A*. *longipes* cx1 and *A*. *alternata* cx2 are 35.7 Mb and 33.5 Mb, covering 89.5% and 89.6% of the predicted genome sizes, respectively (Table 1). These numbers are similar to the published genome sizes of *A*. *alternata* SRC1lrK1f (32.99 Mb) and *A*. *brassicicola* (29.54 Mb). The *A*. *longipes* cx1 genome assembly consists of 2,836 contigs with a N50 of 32.5 kb. The *A*. *alternata* cx2 genome assembly resulted in 1,406 contigs with a N50 of 47.4 kb. The N50 scaffold sizes of *A*. *longipes* cx1 and *A*. *alternata* cx2 were 208.2 kb and 1,889.4 kb, respectively. The GC content is 51.03% and 50.98% for the genome of *A*. *longipes* cx1 and *A*. *alternata* cx2, respectively.

**Table 1:** Statistics for the assembled genome sequences of *A*. *longipes* cx1 and *A*. *alternata* cx2.

	*A*. *longipes* cx1		*A*. *alternata* cx2	
	Contigs	Scaffolds	Contigs	Scaffolds
**N50**	32,505	199,330	47,420	1,889,411
**Max length**	171,230	1,172,862	188,398	5,106,132
**Total length**	35,710,164	36,587,255	33,528,339	33,816,569
**Total number**	2,836	2,854	1,406	540
**Assemblied genome size**	35,710,164		33,528,339	
**Estimated genome size**	39,987,783		37,396,367	
**Coverage (%)**	89.5		89.6	
**GC (%)**	51.03		50.98	

**Fig 4:**
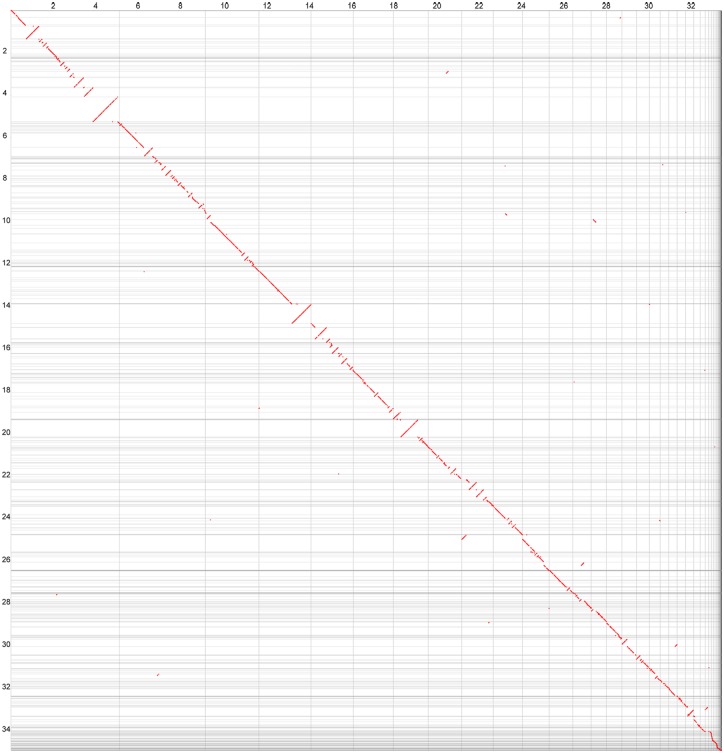
Synteny dotplot of *A*. *longipes* cx1 (y-axis) and *A*. *alternata* cx2 (x-axis). Regions of homology are plotted as diagonal lines.

Evaluation of assembled genomes using Reaper [20] showed that 93.55% bases in the *A*. *longipes* cx1 assembled genome, and 94.49% in the *A*. *alternata* cx2 genome were error free bases. The CEGMA mapping protocol [21] showed that the genome assemblies of *A*. *longipes* cx1 and *A*. *alternata* cx2 captured 99.19% (246 of 248) and 98.39% (244 of 248) complete ultra-conserved core proteins, respectively (S1 Table). These results demonstrate the high quality of both two genome assemblies in this study. Additionally, scaffold alignment using NUCmer in MUMmer3.23 showed that *A*. *longipes* cx1 and *A*. *alternata* cx2 are highly syntenic [22] (Fig 4).

### Genome annotation

Using MAKER2 [23], a total of 12,690 protein-coding genes were predicted in the genome of *A*. *longipes* cx1, and a total of 12,041 in the genome of *A*. *alternata* cx2. These predicted genes accounted for 63.45% and 66.69% of the assembled *A*. *longipes* cx1 and *A*. *alternata* cx2 genomes, with an average gene length of 1,784.89 bp and 1,855.42 bp, respectively (Table 2). The result shows that *A*. *longipes* cx1 has a larger genome and more protein-coding genes than *A*. *alternata* cx2.

**Table 2:** *A*. *longipes* cx1 and *A*. *alternata* cx2 genome features.

General genome features	*A*. *longipes* cx1	*A*. *alternata* cx1
**Size (bp)**	35,710,164	33,528,339
**Repeats percent (%)**	3.07	1.73
**Protein-coding genes**	12,690	12,041
**Percent coding (%)**	63.45	66.69
**Average gene size (bp)**	1,784.89	1,855.42
**Average exon number**	2.82	2.86
**tRNAs genes**	99	98

With functional annotation, about 5,938 (46.79%), 6,133 (48.33%), 6,986 (55.05%) and 10,895 (85.86%) of the predicted genes in *A*. *longipes* cx1, and about 5,825(48.38%), 5,884 (48.87%), 6,724 (55.84%) and 10,311(85.63%) genes in *A*. *alternata* cx2 had homologies with known functions in the following four databases, respectively: the Gene ontology (GO) [24], Kyoto Encyclopaedia of Genes and Genomes (KEGG) [25], SwissProt and TrEMBL databases [26]. In total, there were 10,921 genes in *A*. *longipes* cx1 and 10,336 genes in *A*. *alternata* cx2 identified as common to all four protein databases (S2 Table).

The content of transposable elements (TE) may have profound impacts on the genome rearrangement and synteny loss in fungi [27]. The repeat sequences accounted for 3.07% of the *A*. *longipes* cx1 genome, and 1.73% of the *A*. *alternata* cx2 genome. Among all repeat sequences, TEs made up 2.76% of the *A*.*longipes* cx1 genome, which was almost twice that of the *A*. *alternata* cx2 genome (1.48%) (S3 Table). Because high TE content is a hallmark of CDCs [28], this result implies that the *A*. *longipes* cx1 genome might contain CDCs. Meanwhile, the TE content has a positive correlation with genome rearrangement, suggesting that *A*. *longipes* has a more flexible genome.

For annotation of non-coding genes, we identified 99 tRNAs for *A*. *longipes* cx1, and 98 tRNAs in *A*. *alternata* cx2. In addition, 26 snRNAs, 3 sRNAs and 132 rRNAs were annotated in *A*. *longipes* cx1, and 32 snRNAs, 3 sRNAs and 106 rRNAs were annotated in *A*. *alternata* cx2.

### Phylogenetic analysis

**Fig 5:**
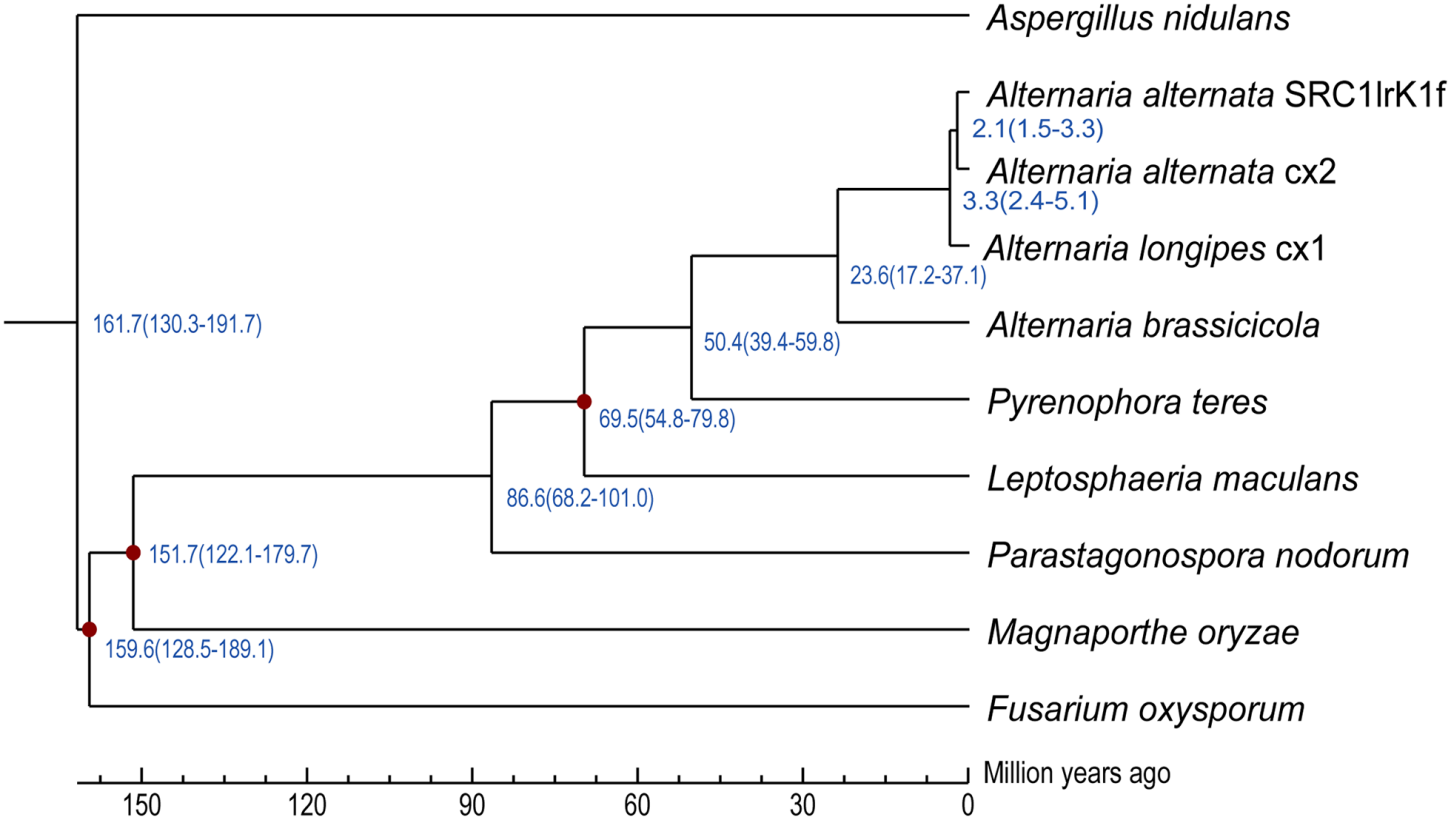
Phylogenetic relationship among *A*. *longipes* cx1, *A*. *alternata* cx2, *F*. *oxysporum*, *M*. *oryzae*, *A*. *nidulans*, *L*. *maculans*, *P*. *teres*, *P*. *nodorum*, *A*. *abrassicicola and A*. *alternata* SRC1lrK1f. The estimates of divergence time and its interval based on sequence identity are indicated at each node. The red dot on branches means divergence time has been adjusted by fossil evidence.

Single copy orthologous genes defined by OrthoMCL [29] were chosen to carry out phylogenetic analysis. Phylogenetic relationship based on 2,702 single copy orthologous genes among *A*. *longipes*, *A*. *alternata*, *Fusarium oxysporum*, *Magnaporthe oryzae*, *Aspergillus nidulans*, *Leptosphaeria maculans*, *Pyrenophora teres*, *Phaeosphaeria nodorum*, *A*. *abrassicicola* and *A*. *alternata* SRC1lrK1f were constructed using MrBayes [30]. The estimated divergence time of 3.3 (2.4–5.1) million years ago (MYA) between *A*. *longipes* cx1 and *A*. *alternata* cx2 (Fig 5) is consistent with the divergence time between species in closely related genera (i.e., 4.1 MYA between *Cochliobolus sativus* and *C*. *heterostrophus*; 7.1 MYA between *P*. *teres* and *P*. *tritici-repentis*) [31], indicating a recent event of speciation. The estimated divergence time between *A*. *alternata* SRC1lrK1f and *A*. *alternata* cx2 is 2.1 (1.5–3.3) MYA, which further suggests that *A*. *alternata* cx2 belongs to the species *A*. *alternata*. Phylogenetic analysis based on single copy orthologous genes further confirmed that *A*. *longipes* cx1 and *A*. *alternata* cx2 belong to two different species.

**Fig 6:**
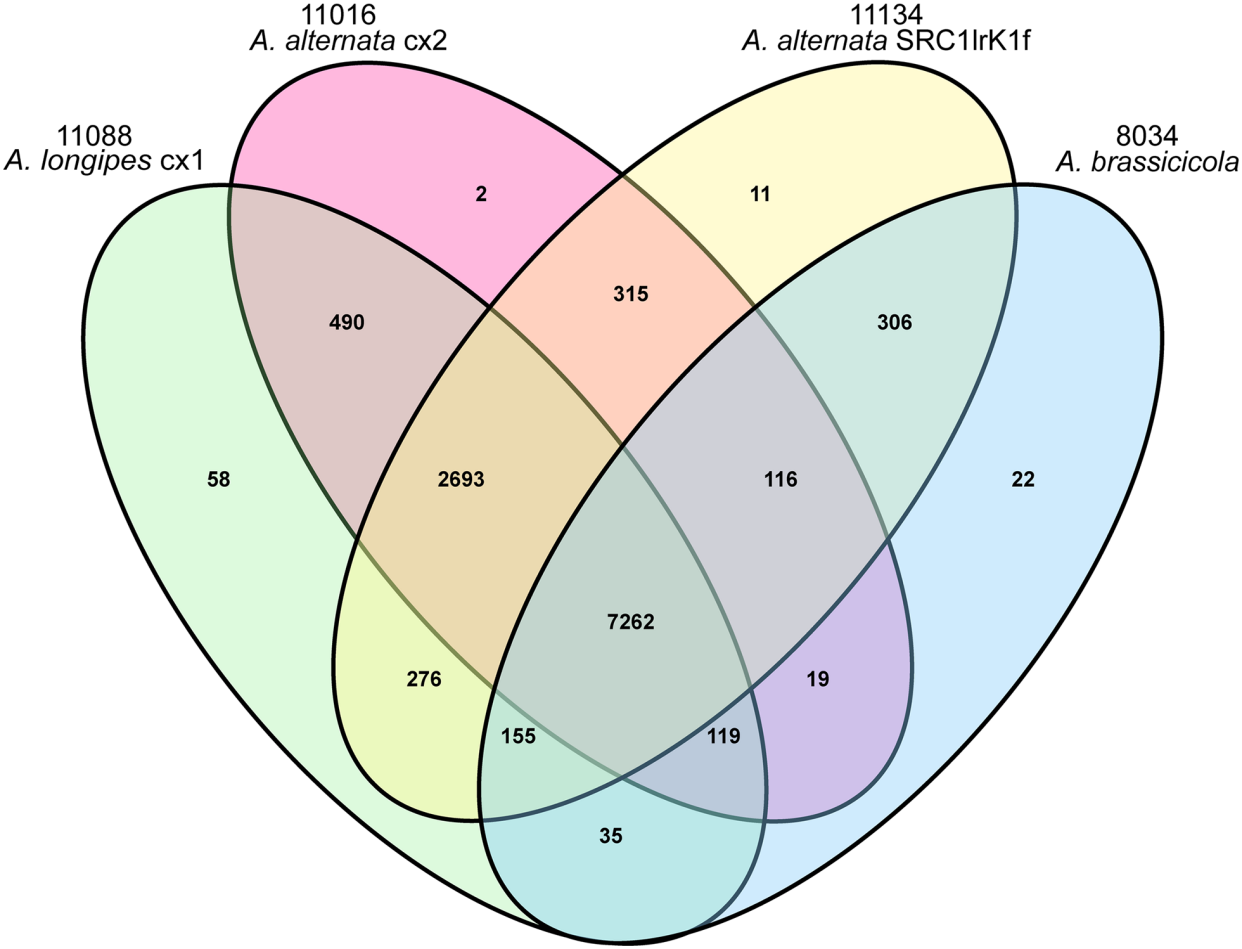
Venn diagram showing the number of unique and shared gene families among *A*. *longipes* cx1, *A*. *alternata* cx2, *A*. *brassicicola and A*. *alternata* SRC1lrK1f.

Based on pair-wise protein sequence similarity, we carried out gene family clustering analysis on all *A*. *longipes* cx1, *A*. *alternata* cx2, *A*. *abrassicicola* and *A*. *alternata* SRC1lrK1f genes using OrthoMCL [29] (Fig 6). 12,690 genes in *A*. *longipes* cx1 and 12,041 genes in *A*. *alternata* cx2 were clustered into 11,088 and 11,016 gene families, respectively. Fifty-eight *A*. *longipes* cx1 specific gene families contained 154 genes, whereas *A*. *alternata* cx2 had 2 specific gene families with 4 genes (S4 Table). Interestingly, *A*. *alternata* cx2 shared more gene families with *A*. *longipes* cx1 (10,983) than with *A*. *alternata* SRC1lrK1f (10,802).

We investigated gene families only shared by *A*. *longipes* cx1 and *A*. *alternata* cx2 and absent in *A*. *brassicicola and A*. *alternata* SRC1lrK1f (S5 Table). They were annotated by KEGG database. For example, six genes in the gene family No. 668 in *A*. *longipes* cx1 and *A*. *alternata* cx2 were annotated as hypothetical glycogen debranching enzymes (Table 3). These enzymes facilitate the breakdown of glycogen, which might help the fungal pathogens catabolize and utilize nutrients in tobacco leaves. The gene family No. 1123 was identified as pentosyltransferases (Pfs), NACHT and WD domain proteins. They might be closely related to carbohydrate metabolism in tobacco. Genes in the gene family No. 8849 were defined as WSC domain protein-coding genes. Previous studies have elucidated that *WSC1*, *WSC2* and *WSC3* genes encode putative receptors that maintain cell wall integrity under heat stress [32]. Moreover, the functional characteristics and cellular localization of WSC suggest that they may mediate intracellular responses to environmental stress in yeast [33].

**Table 3:** Three gene families specific to *A*. *longipes* cx1 and *A*. *alternata* cx2 have different gene copies between these two strains.

	*A*. *longipes* cx1		*A*. *alternata* cx2	
FamilyID	GeneID	KO	GeneID	KO
No. 668	AL_scaffold178_10754	K01196	AA_scaffold30_2734	K01196
	AL_scaffold301_9424	K01196		
	AL_scaffold314_7028			
	AL_scaffold329_1	K01196		
	AL_scaffold481_4249	K01196		
No. 1123	AL_scaffold249_5852	K00777	AA_scaffold30_2732	K00777
	AL_scaffold29_2251	K00777		
	AL_scaffold325_9746	K00777		
	AL_scaffold340_8411	K00777		
No. 8849	AL_scaffold260_3766	K01238	AA_scaffold27_18	K01238
	AL_scaffold422_673	K01238		

Furthermore, *A*. *longipes* cx1 had many unique gene families (S6 Table). Based on the functional annotation, we found that these gene families covered extensive parts of biological processes, including genes in the secondary metabolic pathways (gene family No. 11699) and pathogen-plant interaction (gene family No. 11728 and No. 11792), which proposed that these families may be involved in pathogen-plant interaction during the infection.

### Secondary metabolic pathways (NRPS and PKS)

*Alternaria spp*. produce more than 60 secondary metabolites [34], including important HSTs and non-HSTs that ultimately cause plant cell death. Most of them are versatile compounds of polyketides and non-ribosomal peptides, which are usually generated by non-ribosomal peptide synthase (NRPS) and polyketide synthase (PKS), respectively. More importantly, these genes are probably also involved in the synthesis of siderophores, which assists many pathogens to acquire iron from the host during infection. Therefore, they are good candidates for the investigation of virulence factor and toxin synthesis.

Typically, NRPSs mainly consist of adenylation (A), thiolation (T, also known as PCP for peptidyl carrier protein), and condensation (C) domains [35]. Type I fungal PKSs contain ketosynthase (KS), acyltransferase (AT), and acyl carrier protein (ACP) main domains, along with several optional domains, such as b-ketoacyl reductase (KR), dehydratase (DH) and trans-acting enoyl reductase (ER) domains [36]. To detect secondary metabolite biosynthetic genes and pathways, we employed Secondary Metabolite Unique Regions Finder (SMURF) [37] based on PFAM and TIGRFAM domain content to find the PKS and NRPS genes. Considering the architectures of fungal PKSs are very similar to each other, MUSCLE and FastTree [38] were used to find gene clusters of NRPS and PKS genes, respectively. BlastP [39] were used to search specific genes in these two genomes.

We identified 17 NRPS genes and 13 PKS genes in *A*. *longipes* cx1, and 13 NRPS genes and 12 PKS genes in *A*. *alternata* cx2, respectively (S7 Table). Further study was conducted by using antiSMASH [40] online to explore the structural domains of the candidate genes, and unravel their specific roles in the syntheses of secondary metabolites and toxins.

**Fig 7:**
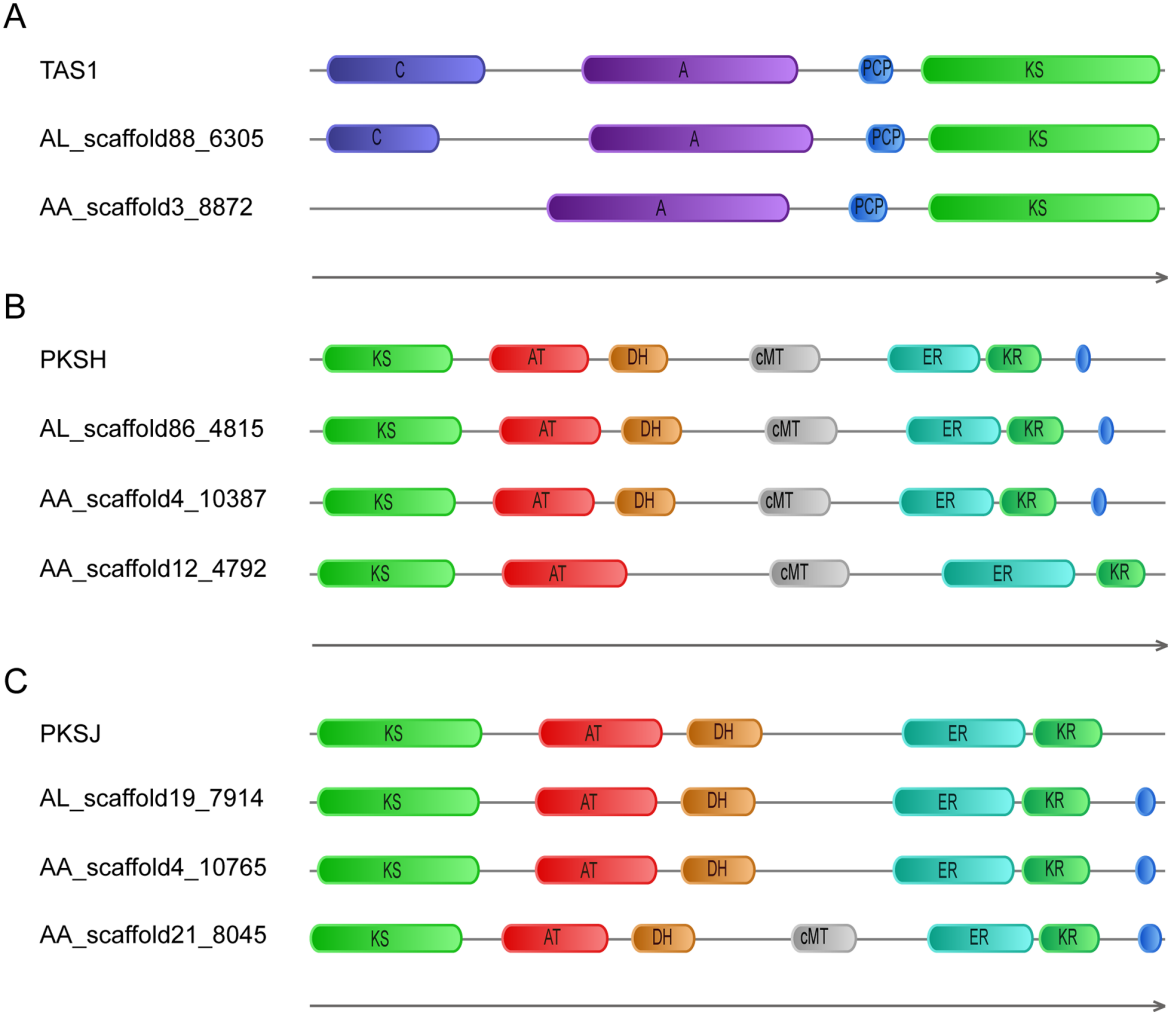
NRPS-PKS homologs in *A*. *longipes* cx1 and *A*. *alternata* cx2. (A) TAS1 homologous gene in *A*. *longipes* cx1 (AL_scaffold88_6305) and *A*. *alternata* cx2 (AA_scaffold3_8872) might be required for TeA synthesis. (B) PKSH homologous gene in *A*. *longipes* cx1 (AL_scaffold86_4815) and *A*. *alternata* cx2 (AA_scaffold4_10387, AA_scaffold12_4792). (C) PKSJ homologous gene in *A*. *longipes* cx1 (AL_scaffold19_7914) and *A*. *alternata* cx2 (AA_scaffold4_10765, AA_scaffold21_8045). The direction of arrow indicates N-terminal.

TeA is a well-known mycotoxin produced by various plant pathogenic fungi, including *Alternaria* spec., *M*. *oryzae* and *Phoma sorghina* [34]. TeA is one of the most toxic *Alternaria* toxins. A recent report revealed that TAS1 in *M*. *oryzae*, a NRPS-PKS hybrid enzyme of 1,602 amino acids, was responsible for TeA synthesis from isoleucine and acetoacetyl-coenzyme A [41]. TAS1 consists of C, A and PCP domains in the NRPS portion, and a KS domain in the PKS portion. This study also verified that the KS domain is responsible for the final cyclization step and the C domain is responsible for the condensation reaction in TeA production by ultra performance liquid chromatography (UPLC) analysis of metabolites. As Fig 7A illustrates, gene AL_scaffold88_6305 and gene AA_scaffold3_8872 were considered as *TAS1* homologs, suggesting a potential role in the production of TeA. Gene AL_scaffold88_6305 in *A*. *longipes* cx1 has the same domains with *TAS1* gene, while gene AA_scaffold3_8872 in *A*. *alternata* cx2 lacks of a C domain at the amino terminus, suggesting that it might have a defect in TeA production. Their protein sequences were provided in S3 Text. Interestingly, this is in accordance with previous reports that some isolates of *A*. *alternata* might not produce TeA [8].

Using the same method, we also found genes required for AOH and AWE syntheses. It is well known that one of the postulated core enzymes in the biosynthesis of AOH and AME is PKS. Debjani *et al*. found that the timing of the expression of two PKS genes, *pksJ* (JX103645) and *pksH* (JX103643) are correlated with the production of AOH and AME [9]. AL_scaffold86_4815 and AA_scaffold4_10387 shared high homology with *pksH*, and AL_scaffold19_7914 and AA_scaffold4_10765 shared high homology with *pksJ* (Fig 7B and 7C). Combined with gene cluster results, we found an additional copy of *pksH* homolog (AA_scaffold12_4792), and an additional copy of *pksJ* homolog (AA_scaffold21_8045) in *A*. *alternata* cx2. AA_scaffold12_4792 protein only lacks a DH domain. AA_scaffold21_8045 protein is almost the same as *pksJ* with an additional cMT domain. These data imply gene duplications in *A*. *alternata* cx2 during the evolution process.

NRPS or PKS genes responsible for synthesis of HSTs generally reside on CDCs. However, it is a big challenge for us to find genes responsible for AT-toxin synthesis because of the insufficient research up to now. To figure out CDCs in *A*. *longipes* cx1 and *A*. *alternata* cx2, two CDC marker gene on *A*. *arborescens* (tomato pathotype), *ALT1*, a PKS gene involved in AAL toxin biosynthesis, and *AaMSAS* gene, a putative 6-MSA-type PKS gene [28, 42] were used as query to BLAST in *A*. *longipes* cx1 and *A*. *alternata* cx2 genome. However, there is no gene homologous to *ALT1* in both genomes. What’s interesting is that two genes, AL_scaffold266_5850 and AL_scaffold337_4970, identical to *AaMSAS* were found in *A*. *longipes* cx1, while none was found in *A*. *alternata* cx2 (S2 Fig). The results imply that scaffold226 (18 Kb) and scaffold 337 (11 Kb) in *A*. *longipes* cx1 on which *AaMSAS* homologous gene reside might be fragments of CDCs.

### Plant pathogen interaction

Searching against pathogen host interaction database (PHI-base) [43] identified 2,180 (17.18%) genes in *A*. *longipes* cx1 and 2,063 (17.13%) genes in *A*. *alternata* cx2 that might be involved in pathogenicity and virulence pathways. Among the proteins that showed over 70% identity with proteins in the PHI-base, 50 of 122 matches in *A*. *longipes* cx1 and 52 of 116 matches in *A*. *alternata* cx2 were labeled “loss of pathogenicity or reduced virulence” as the phenotype characteristic in mutant strains (S8 Table).

To study genes in the plant-pathogen interaction, we searched each genome using the KEGG Orthology (KO) number in the KEGG plant-pathogen interaction pathway (ko04626). As the result showed, *A*. *longipes* cx1 has more genes involved in this pathway (Table 4). For the calmodulin gene (K02183) family, there are 8 genes in *A*. *longipes* cx1, and 5 genes in *A*. *alternata* cx2. These genes potentially regulate the biological activities of many cellular proteins and transmembrane ion transporters mainly in a Ca^2+^-dependent manner in fungi. The increase of cytosolic Ca^2+^ concentration in plants is a regulator for the production of reactive oxygen species and the localized programmed cell death/hypersensitive response [44]. The higher number of calmodulin genes existed in *A*. *longipes* cx1 than *A*. *alternata* cx2 may imply stronger Ca^2+^ storage and release capacity.

**Table 4:** Genes related to KEGG plant-pathogen interaction.

	*A*. *longipes* cx1	*A*. *alternata* cx2
KO	GeneID	GeneID
K00864	AL_scaffold122_10370	AA_scaffold14_4293
K02183	AL_scaffold28_2655	AA_scaffold7_9519
	AL_scaffold5_7042	AA_scaffold8_5177
	AL_scaffold553_987	AA_scaffold1_866
	AL_scaffold688_9778	AA_scaffold10_11716
	AL_scaffold812_594	AA_scaffold10_11716
	AL_scaffold893_5442	
	AL_scaffold97_3815	
	AL_scaffold14_5990	
K04079	AL_scaffold5_7293	AA_scaffold3_8231
	AL_scaffold522_3984	AA_scaffold10_11537
	AL_scaffold539_259	
	AL_scaffold65_4222	
K12795	AL_scaffold18_10272	AA_scaffold11_2880
	AL_scaffold124_9768	

### Carbohydrate degrading enzymes

**Fig 8:**
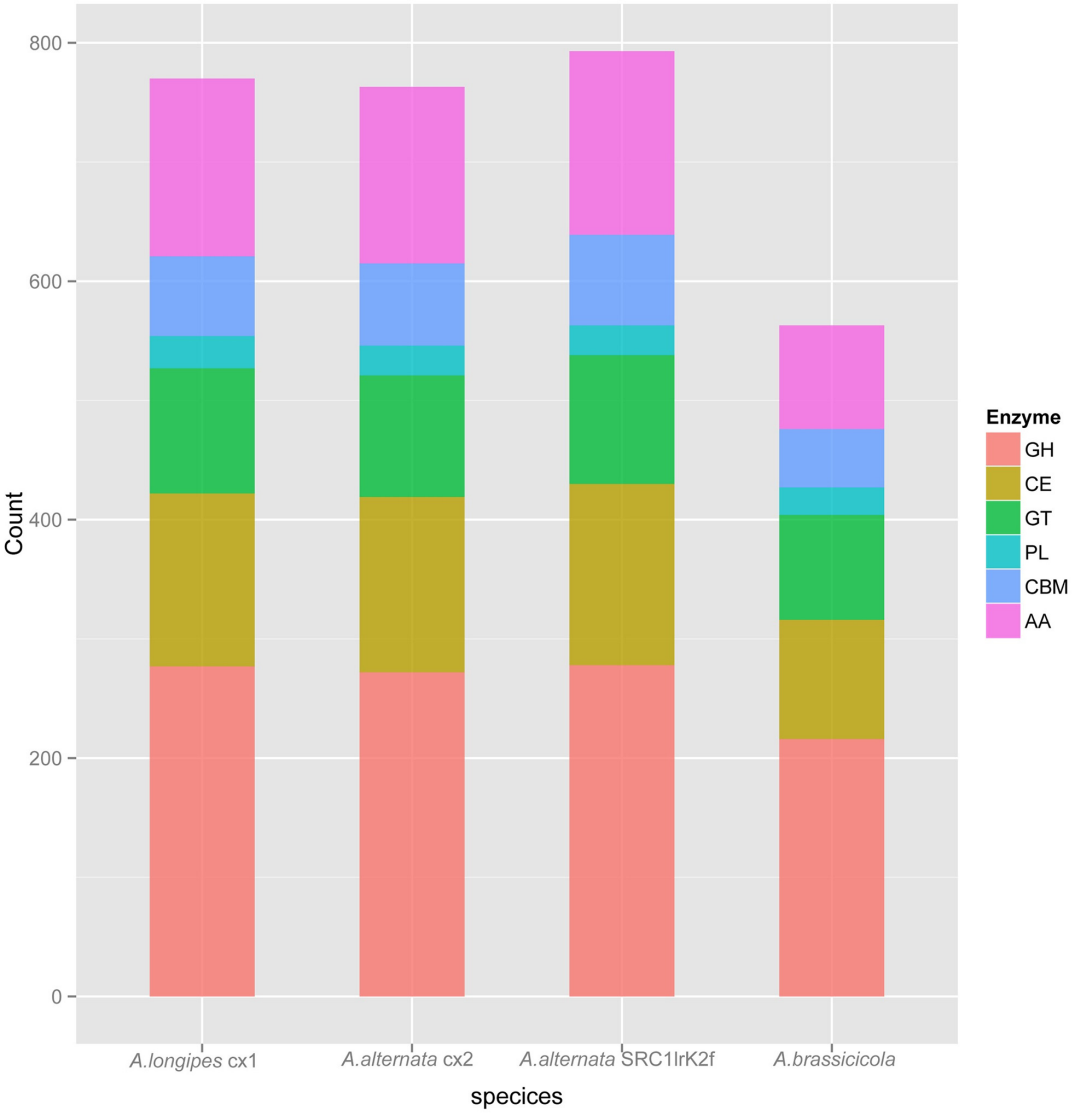
Distribution of carbohydrate-active enzymes gene families in *A*. *longipes* cx1, *A*. *alternata* cx2, *A*. *abrassicicola* and *A*. *alternata* SRC1lrK1f.

Carbohydrate-active enzymes (CAZymes) are responsible for the breakdown, biosynthesis or modification of glycoconjugates, oligo- and polysaccharides. Besides making energy harvest from the plant tissues possible, pathogen CAZymes play a central role in the degradation of plant cell wall, the penetration into the host tissue, and the host-pathogen interactions [45]. CAZymes are grouped into four functional classes based on their catalytic modules or functional domains: glycoside hydrolases (GHs), glycosyltransferases (GTs), polysaccharide lyases (PLs), and carbohydrate esterases (CEs) [45]. To investigate CAZymes composition in both *A*. *longipes* cx1 and *A*. *alternata* cx2 genomes, HMMScan was used to search each predicted fungal proteomes against dbCAN (release 3.0) CAZymes database [46]. As shown in Fig 8, *A*. *longipes* cx1 genome encoded 554 putative CAZymes, including 277 GH, 105 GT, 145 CE, and 27 PL. *A*. *alternata* cx2 contained 546 putative CAZymes, including 272 GH, 102 GT, 147 CE, and 25 PL. Among these four classes, CE, GH, and PL classes are considered as cell wall degrading enzymes (CWDE) due to their roles in the disintegration of the plant cell wall by bacterial and fungal pathogens [47, 48]. However, the numbers of genes in CE, GH, and PL classes did not differ between *A*. *longipes* cx1 and *A*. *alternata* cx2 (S9 Table).

### Secretome prediction

The secretome is defined as the global set of proteins produced by a cell and exported to the extracellular space in a determined time and condition [49]. The secretome plays an important role in the interactions with the environment, degradation of complex organic compounds, and in modulating directly or indirectly pathogen-host interactions. To compare the difference between *A*. *longipes* cx1 and *A*. *alternata* cx2, SignalP [50] and TargetP [51] were used to detect protein sequences with signal peptides, and TMHMM [52] was used to detect transmembrane helixes. As a result, 899 secreted proteins in *A*. *longipes* cx1 and 865 secreted proteins in *A*. *alternata* cx2 were predicted. Among all secreted protein encoding genes in *A*. *longipes* cx1, 212 genes were identified as cell wall degrading enzymes. In comparison, 206 genes in secreted protein encoding genes of *A*. *alternata* cx2 belonged to cell wall degrading enzymes. In addition, 8 genes in *A*. *longipes* cx1 and 8 genes in *A*. *alternata* cx2 were annotated as “effector” by BLASTN with PHI-base (S10 Table), and the “effector” were reported to be required for the direct or indirect recognition of a pathogen only in resistant host genotype [43, 53].

## Conclusion

In this study, we reported two high quality genomes of *Alternaria* pathogens. Through the large-scale genomic analysis, we found NRPS and PKS genes in both genomes that likely participate in TeA, AOH and AME synthesis. It is interesting that *A*. *longipes* cx1 possessed more NRPS and PKS genes in total, while *A*. *alternata* cx2 gained another copy of PKS genes responsible for AOH and AME synthesis. By comparative genomic analysis, we found more genes with a putative function in pathogen-plant interaction, more carbohydrate degrading enzymes and more secreted proteins in *A*. *longipes* cx1 than in *A*. *alternata* cx2.

In summary, our results provide a novel perspective for studying the synthesis of various toxins and complex interactions with the hosts. It establishes a powerful basis for further identification of genes involved in AT-toxin synthesis. Conceivably, these resources will improve the understanding of important pathogens of the genus *Alternaria*, increase the genome information for pathogenic fungi, and facilitate the study of pathogenicity mechanisms of HSTs and various mycotoxins.

## Materials and Methods

### Strain acquiring and identification

Two different isolates were isolated from different infected tobacco cultures using single fungal spore separating method. First, the spores were scraped carefully from the brown spots lesions on tobacco leaves by a blade. CX1 was separated from typical brown spot lesions on tobacco leaves, while CX2 was isolated from brown spot lesions on sunburned tobacco leaves that are larger than typical brown spot lesions. Spores were diluted in sterile water to a density of about 300 spores/ml. The spore suspension was subsequently spread uniformly on a PDA plate containing ampicillin (950 μg/ml), rifampicin (100 μg/ml) and quintozene (50 μg/ml) to ensure that single spores grew isolated. Germinated spores were excised from the PDA plate and transferred to a fresh plate.

For strain identification, ITS sequences were obtained by PCR using primer pairs (ITS5 and ITS4, ITS1 and ITS4, ITS3 and ITS4, and ITS1and ITS2) (S11 Table). Phylogenetic relationship according to ITS was analyzed by using MEGA5 among CX1, CX2, *A*. *longipes* EGS30-033 (accession: AY751457.1), *A*. *alternata* SDHeze-9 (accession: KT238888.1), *A*. *tenuissima* CSPF5 (accession: KU508797.1) and *A*. *brassicicola* Ab4UP (accession: KF542552.1) from NCBI.

To confirm the strain identification, phylogenetic analysis based on large co-linear sequence was implemented. Scaffolds about 200 kb in length in CX1 were selected and used as query to search the matching sequence of other fungi in *Alternaria* Genomes Database by BLASTN on their website. Finally, six scaffolds of about 195 kb from CX1 (scaffold45), CX2 (part of scaffold9), *A*. *longipes* bmp0313 (part of contig ALGCTG00140), *A*. *alternata* atcc11680 (part of contig ATNCTG00647), *A*. *alternata* atcc66891 (part of contig AATCTG00103) and *A*. *tenuissima* bmp0304 (part of contig AT2CTG00134) were chosen to construct phylogenetic tree using MEGA5.

### Sequencing

Genomic DNA was extracted from fresh fungal hyphae by cetyltrimethyl ammonium bromide (CTAB) method [54]. In brief, fungal hyphae were ground in liquid nitrogen, and CTAB buffer was added to breakdown the cell wall. Mixture of phenol, chloroform and isoamyl alcohol was used to extract DNA and isopropyl alcohol was used to precipitate DNA. The DNA sediment was then washed with 75% ethanol and dissolved in sterile water.

To build small insert libraries, 2 μg of DNA were sheared to fragments of 300–1100 bp, end-repaired, A-tailed and ligated to Illumina paired-end adapters (Illumina). The ligated fragments were size selected at 427, 603 and 1042 bp for *A*. *longipes* cx1 and 430, 680 and 820 bp for *A*. *alternata* cx2 on agarose gel and amplified by PCR to yield the corresponding short insert libraries. All these DNA libraries were sequenced on the Illumina HiSeq 2500 platform. In total, we generated 277.4 M of usable sequence for *A*. *longipes* cx1 and 180.0 M for *A*. *alternata* cx2, respectively.

### Genome assembly

First, a stringent filter and correction processing was carried out. All reads were removed with duplications and adapters and subsequently preprocessed by filtering out reads with more than 30 low-quality bases or more than 5% unknown bases. The sequence errors were corrected based on *K-mer* frequency information using a script named Corrector_HA (versoin 2.01) made by the Beijing Genomics Institute (BGI). For both *A*. *longipes* cx1 and *A*. *alternata* cx2 genome assemblies, we chose K = 17 bp, and corrected sequencing errors for the *17-mer*s with a frequency lower than 3.

We used *17-mer* analysis to evaluate the genome size and heterozygosity. All *17-mer* sequences were extracted from paired-end reads from short insert size libraries (< 1 kb) after filter and correction, and the frequency of each *17-mer* was calculated and plotted. The genome size G = K_num/Peak_depth, where the K_num is the total number of *17-mer*, and Peak_depth is the expected value of *17-mer* depth.

We assembled the short reads using JR-Assembler [55]–an extension-based *de novo* assembler. It runs in five steps: raw read processing, seed selection, seed extension, repeat detection, and contig merging by SSAKE based Scaffolding of Pre-Assembled Contigs after Extension (SSPACE) [56]. We chose the same optimized parameters (minOverlap 30, maxOverlap 40, Assembly Read Length 87) for both genome assemblies.

To confirm the genome assembly, Reaper was performed to evaluate the assembled genome quality with default parameters, which is a tool that precisely identifies errors in genome assemblies [20]. Meanwhile, software CEGMA [21] with default setting was also used to estimate the sequence completeness of the assembly.

The assembled scaffolds of these two genomes were aligned using NUCmer in MUMmer3.23 with the minimum length of a cluster of matches set by 100 (c = 100) [22]. The assembled genome with longer scaffolds (*A*. *alternata* cx2) was used as the reference, while the genome of *A*. *longipes* cx1 was used as the query. After merging adjacent alignments with gaps less than 300 bp, alignments shorter than 10 kb were discarded. Subsequently, all alignments were output in the reference’s order and a dot-plot SVG graph was generated by a perl script.

### Repeat annotation

For the identification of known TEs in the genome assembly, we employed RepeatMasker (version 3.3.0) [57] against the Repbase 16.0 [58] TE library, and then executed RepeatProteinMask in RepeatMasker package to identify TEs by aligning the genome sequence to a self-taken curated TE protein database. We also constructed a *de novo* repeat library using Piler, RepeatScount [59] and LTR_FINDER [60], followed by filtering sequences less than 100 bp. The generated results were consensus sequences and classification information for each repeat family. Then we used RepeatMasker again on the library built in the above steps. For tandem repeat prediction, we used RepeatMasker with the “-noint” option, including simple repeat, satellites, and low complexity repeats. TRF [61] were also used to predict tandem repeats, with parameters set to “Match = 2, Mismatch = 7, Delta = 7, PM = 80, PI = 10, Minscore = 50, and MaxPeriod = 12”.

### Protein-coding gene prediction

To predict protein-coding genes, we employed the MAKER pipeline [23] in default parameters. MAKER2 executes *ab inito* prediction using the programs SNAP, Augustus, and GeneMark-ES, and evidence-based annotation using EST and protein homology as references. Proteins from *L*. *maculans*, *P*. *nodorum*, *P*. *teres*, *Pyrenophora triticirepentis*, *A*. *brassicicola* and transcripts of the published genome of *A*. *alternata* SRC1lrK1f were used as references in the homology-based annotation.

### Functional gene annotation

To assign preliminary GO terms to the protein-coding genes, InterProScan (version 5) [62] was used to screen predicted proteins against publicly available databases including Pfam, PRINTS, PROSITE, ProDom, PANTHER and SingnalP. The KEGG Orthology database, Uniprot/SwissProt and UniProt/TrEMBL database were searched for homology-based function assignments by blastall (e-value ≤ 1e-5). Potential pathogenicity factors were identified through scanning protein-coding genes in the PHI-base using an e-value of 1e-5 and ≥ 50% coverage as criteria.

### Non-coding RNA annotation

Genes encoding tRNAs were identified using tRNAscan-SE with appropriate default parameters. The rRNA fragments were identified by aligning the rRNA template sequences from the yeast genome using BlastN at an e-value of 1e-5. Other ncRNAs, including miRNA, sRNA and snRNA, were identified using INFERNAL-1.1 [63] software by searching against the Rfam database with appropriate parameters (—rfam,—cut_ga,—nohmmonly).

### Gene family cluster identification

The OrthoMCL (orthomclSoftware-v2.0.9) [29] was used to define a gene family as a group of orthologs or in-paralogs. For genes with alternative spliced variants, the longest transcript was used to represent the gene. To identify gene family clusters in these species, all-versus-all protein searches were performed using BLASTP with an e-value of 1e-5. The homologous segment pairs were processed using the OrthoMCL with an e-value cutoff of 1e-5, and MCL (mcl-14-137) was used to define final orthologs and paralogs with an inflation value of 1.5.

### Phylogenetic analysis

Single copy gene family genes (i.e., one copy in all species) were used to construct a phylogenetic tree of the *F*. *oxysporum*, *M*. *oryzae*, *A*. *nidulans*, *L*. *maculans*, *P*. *teres*, *P*. *nodorum*, *A*. *abrassicicola* and *A*. *alternata* SRC1lrK1f. Multiple sequence alignments were performed using MUSCLE (muscle-3.8.31) [64]. Four-fold degenerate sites were extracted from each gene and concatenated into a supergene for each species. At last, MrBayes software (http://mrbayes.sourceforge.net, version 3.1.2) [30] was used to reconstruct the evolutionary relationships between species.

### Divergence time estimation

We performed divergence time estimation with r8s, which estimates absolute rates ("r8s") of molecular evolution and divergence times on a phylogenetic tree, and then used the MCMCTREE program [65], implemented in the PAML package to estimate divergence times. Calibration time for the common ancestor from the TimeTree database (http://www.timetree.org/) was used to calibrate the divergence time estimation.

### Secretome prediction

TargetP [51] was used to identify signal peptides and predict the subcellular location of proteins. Then SignalP [50] was used to predict the presence and location of signal peptide cleavage sites. These proteins were subsequently scanned for the presence of transmembrane helixes using the hidden Markov model topology predictor TMHMM [52]. Proteins with signal peptide and lacking transmembrane domains were deemed secreted proteins. The proteins with a length of less than 200 amino acids or with a cysteine content of less than 1.5% were removed and the candidate effectors were obtained.

## Supporting Information

S1 Fig *17-mer* frequency distribution of sequencing reads. (A) One sequencing library with insert size of 916 bp of *A*. *longipes* cx1. (B) One sequencing library with insert size of 694 bp of *A*. *alternata* cx2.
(PDF)

S2 Fig Two scaffolds seem like CDCs in *A*. *longipes* cx1. (PDF)

S1 Table Statistics of the completeness of assembled *A*. *longipes* cx1 and *A*. *alternata* cx2 genome based on 248 CEGs. (A) CEGMA report of *A*. *longipes* cx1. (B) CEGMA report of *A*. *alternata* cx2.
(XLSX)

S2 Table Number of genes in *A*. *longipes* cx1 and *A*. *alternata* cx2 with homologs or functional classification in different databases. (XLSX)

S3 Table Statistics of repeat sequences and transposable elements of *A*. *longipes* cx1 and *A*. *alternata* cx2 genomes. (XLSX)

S4 Table Summary of gene family clustering. (XLSX)

S5 Table Gene families only shared by *A*. *longipes* cx1 and *A*. *alternata* cx2. (XLSX)

S6 Table Unique gene families in *A*. *longipes* cx1. (XLSX)

S7 Table List of NRPS and PKS genes in *A*. *longipes* cx1 and *A*. *alternata* cx2. (XLSX)

S8 Table Genes affect the outcome of pathogen-host interaction. (A) Genes in *A*. *longipes* cx1. (B) Genes in *A*. *alternata* cx2.
(XLSX)

S9 Table Statistics of CAZyme genes grouped in CE, GH, and PL classes. (XLSX)

S10 Table Genes annotated as "effector" by PHI-base. (XLSX)

S11 Table Primer pairs used for amplify ITS sequences by PCR. (XLSX)

S1 Text ITS qequence of *A*. *longipes* cx1 and *A*. *alternata* cx2. (TXT)

S2 Text Six large co-linear sequences about 195 kb in length among CX1, CX2, *A*. *longipes* bmp0313, *A*. *alternata* atcc11680, *A*. *alternata* atcc66891 and *A*. *tenuissima* bmp0304. (TXT)

S3 Text Protein sequences of NRPS-PKS homologs in *A*. *longipes* cx1 and *A*. *alternata* cx2. (TXT)
