# Supplementary figures and images for: Comparative Genomics of Pathogens Causing Brown Spot Disease of Tobacco: Alternaria longipes and Alternaria alternata
Source: PLoS One. 2016 May 9;11(5):e0155258. doi: 10.1371/journal.pone.0155258 (PMC4861331; doi:10.1371/journal.pone.0155258)

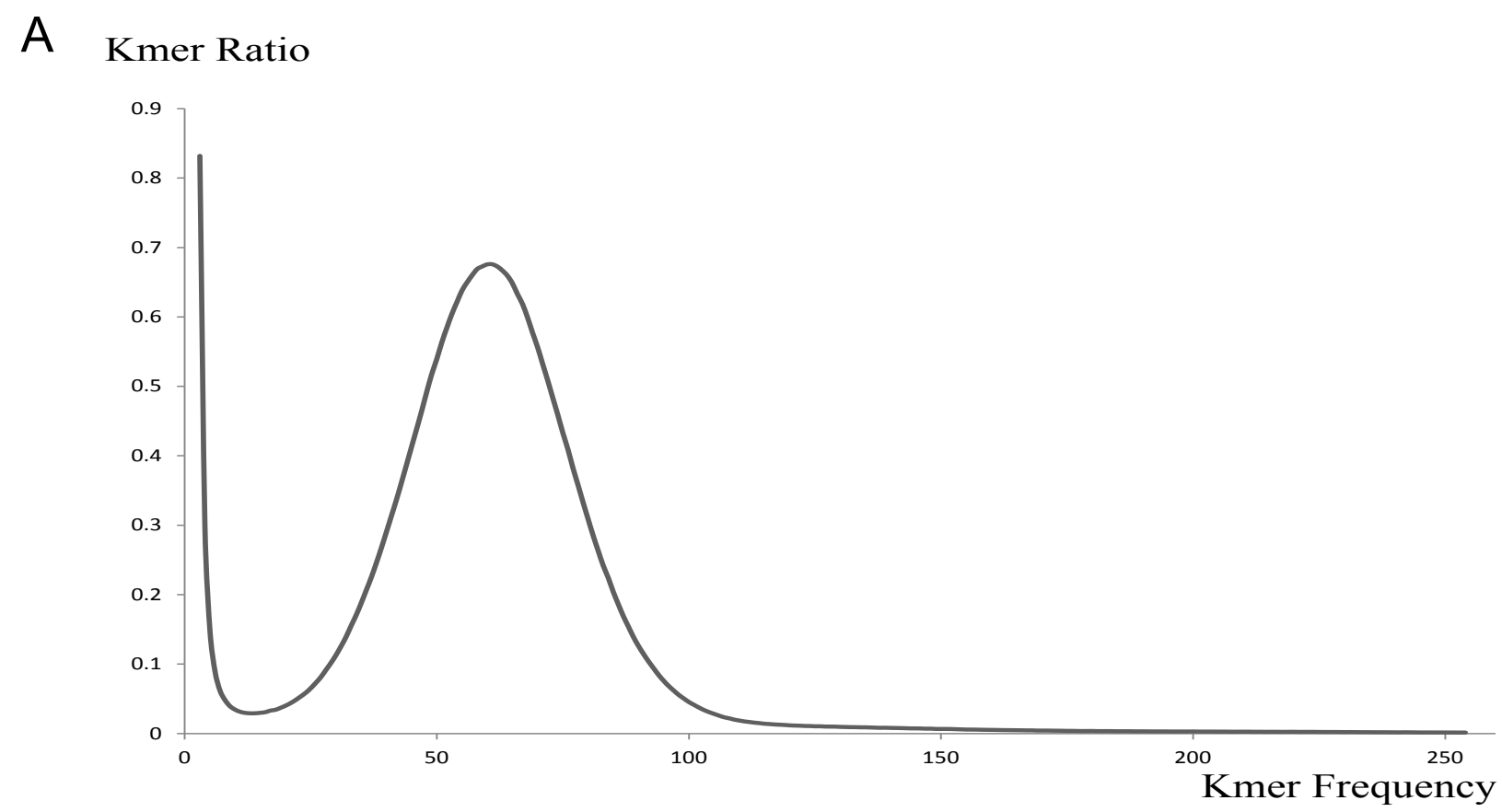

*A. longipes*  
Insert length = 916bp

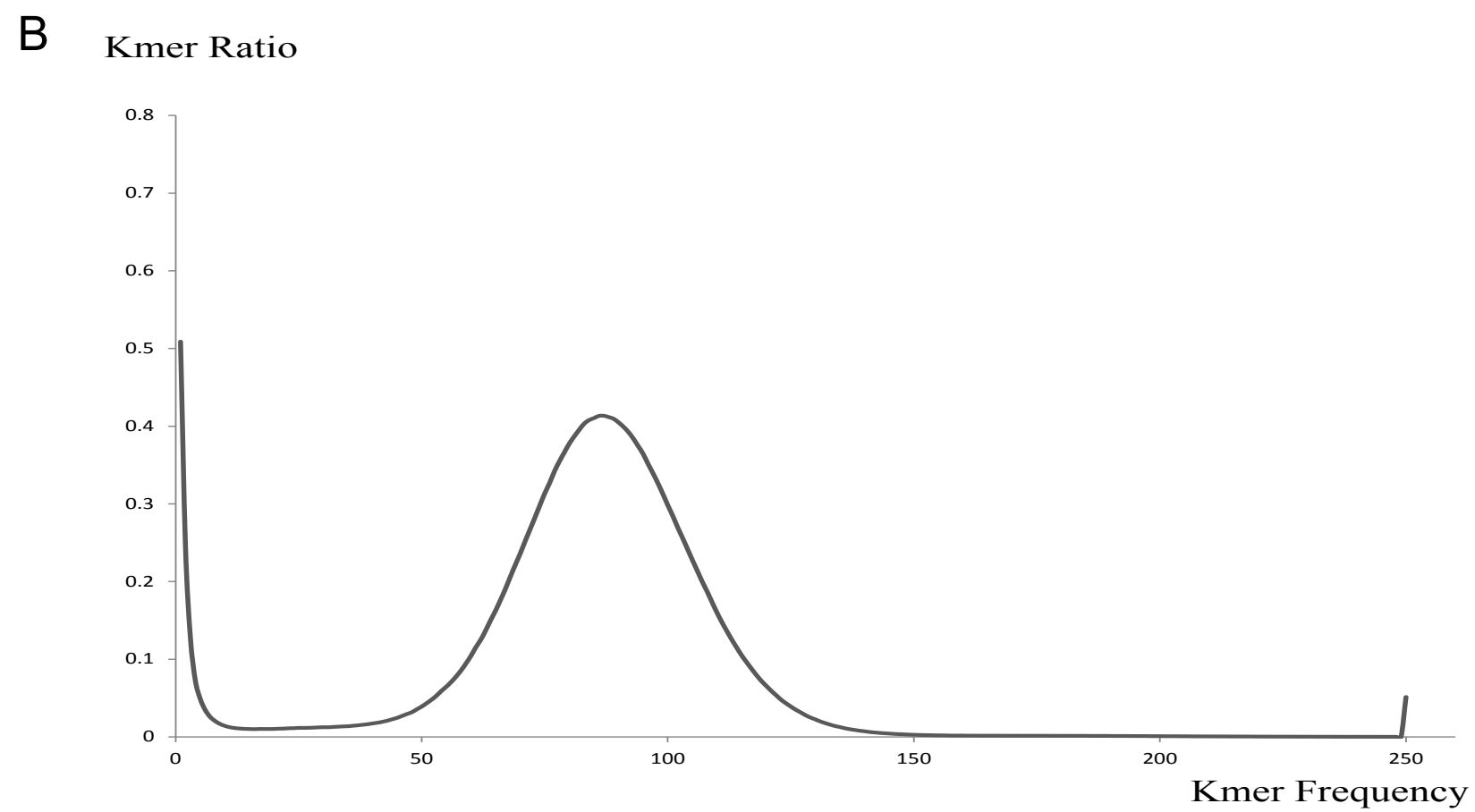

*A. alternata*  
Insert length = 694bp

Supplement: S1 Fig — (A) One sequencing library with insert size of 916 bp of A. longipes cx1. (B) One sequencing library with insert size of 694 bp of A. alternata cx2. (PDF) [file pone.0155258.s001.pdf]

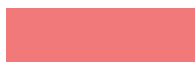

AaMSAS

scaffold 266

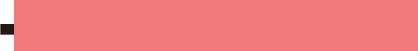

AL\_scaffold266\_5850

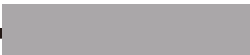

AL\_scaffold266\_5849

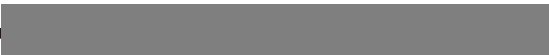

AL\_scaffold266\_5851

19 Kb

scaffold 337

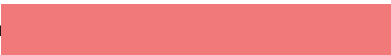

AL\_scaffold337\_4970

11 Kb

Supplement: S2 Fig — (PDF) [file pone.0155258.s002.pdf]
